# Adherence to a Mediterranean-Style Dietary Pattern and Cancer Risk in a Prospective Cohort Study

**DOI:** 10.3390/nu13114064

**Published:** 2021-11-13

**Authors:** Ioanna Yiannakou, Martha R. Singer, Paul F. Jacques, Vanessa Xanthakis, R. Curtis Ellison, Lynn L. Moore

**Affiliations:** 1 Department of Medicine, Preventive Medicine and Epidemiology, Boston University School of Medicine, Boston, MA 02118, USA; ioannay@bu.edu (I.Y.); msinger@bu.edu (M.R.S.); Vanessax@bu.edu (V.X.); ellison@bu.edu (R.C.E.); 2 Graduate Programs in Nutrition and Metabolism, Boston University School of Medicine, Boston, MA 02118, USA; 3 Nutritional Epidemiology, Jean Mayer USDA Human Nutrition Research Center on Aging at Tufts University, Boston, MA 02111, USA; paul.jacques@tufts.edu; 4 Department of Biostatistics, Boston University School of Public Health, Boston, MA 02118, USA

**Keywords:** cancer, Mediterranean diet, diet patterns, cohort study, epidemiology

## Abstract

A Mediterranean-style diet is a healthy eating pattern that may benefit cancer risk, but evidence among Americans is scarce. We examined the prospective association between adherence to such a diet pattern and total cancer risk. A Mediterranean-style dietary pattern (MSDP) score was derived from a semi-quantitative food frequency questionnaire at exam 5 (1991–1995). Subjects included 2966 participants of the Framingham Offspring Study who were free of prevalent cancer. Cox proportional hazards regression models were used to estimate hazard ratios (HRs) and 95% confidence intervals (CIs), adjusting for demographic, lifestyle, and anthropometric measures. Cox-models were also used to examine effect modification by lifestyle and anthropometric measures. During 18 years of median follow-up, 259 women and 352 men were diagnosed with cancer. Women with moderate or higher adherence to the MSDP had ≥25% lower risks of cancer than women with the lowest MSDP (HR (moderate vs. lowest): 0.71, 95% CI: 0.52–0.97 and HR (highest vs. lowest): 0.74; 95% CI: 0.55–0.99). The association between MSDP score and cancer risk in men was weaker except in non-smokers. Beneficial effects of the MSDP in women were stronger among those who were not overweight. In this study, higher adherence to MSDP was associated with lower cancer risk, especially among women.

## 1. Introduction

The American Institute for Cancer Research (2018) report [1] recommends a healthy dietary pattern rich in fruits, vegetables, legumes, and whole grains while limiting consumption of added sugars and red and processed meats. Many of these recommendations are consistent with a Mediterranean-style diet, which has been suggested in the United States (US), starting with the 2015–2020 Dietary Guidelines, as a healthy eating pattern [2]. The Mediterranean diet has been long described as a well-balanced diet with a predominance of plant-based food sources. However, the specific foods consumed are somewhat variable across different Mediterranean cultures [3,4,5]. The diet in Crete prior to1960 is often considered the model for a traditional Mediterranean diet. It is characterized by higher intakes of vegetables, fruits, legumes, nuts, and non–refined cereals and grain products. Fish and poultry are consumed in moderation, whereas red and processed meats, dairy products, refined grains, and sweets are limited. Olive oil and olives are the most common sources of fat. Red wine is consumed in moderation during meals, and the population is generally quite physically active [5,6]. 

For nearly three decades, epidemiologic evidence has supported the health benefits of adherence to a Mediterranean diet in the primary and secondary prevention of non–communicable chronic diseases [3,7,8]. Although most cohort studies suggest a protective association between higher adherence to a Mediterranean-style diet and risk of specific cancers [9,10,11,12], evidence of its effects on total cancer risk is very limited. Because a Mediterranean-style diet has much in common with an anti-inflammatory diet, it is possible that the diet pattern may have more generalized beneficial effects on cancer risk [13]. Two previous prospective analyses were conducted within the European Prospective Investigation into Cancer and Nutrition cohort (EPIC), but the results were somewhat inconsistent [14,15]. In the first, higher adherence to a Mediterranean diet was strongly associated with a lower cancer risk among Greek women and a lower overall risk of non-smoking-related cancers in both men and women [14]. In the second study, conformity to a Mediterranean diet was protective against cancer occurrence in both Mediterranean and non-Mediterranean countries, although these results were weaker than those in the Greek cohort, and no sex-specific differences were observed [15]. 

Mediterranean diet studies sometimes differ in the means by which the diet pattern is scored. Many studies, including EPIC, have used a Mediterranean diet score based on whether the participant had higher or lower intakes of the relevant foods and nutrients, defined as being above or below the sex-median intakes in that population [16]. Because the actual intakes of these Mediterranean-style foods and nutrients differ widely between population groups, the scores may not uniformly reflect higher adherence to a traditional Mediterranean-style diet as defined by the diet pyramid. A new Mediterranean-style dietary pattern (MSDP) score that does not rely on median intakes was developed in 2009 using data from the Framingham Study [17]. We will use this score to assess adherence to the MSDP.

The primary aim of this prospective study was to examine the longitudinal association between adherence to the MSDP and total cancer risk in the Framingham Offspring Study (FOS) cohort. We examined whether this association was modified by anthropometric measures of body fat or lifestyle factors. In secondary analyses, we assessed the association between each food group considered in the MSDP and total cancer risk. 

## 2. Materials and Methods

### 2.1. Study Population

In 1971, 5124 individuals were enrolled in the prospective FOS. The participants were the children of those who were part of the original Framingham Heart Study. The examination visits, starting with exam 2, were carried out at approximately 4-year intervals [17]. Food frequency questionnaires were used to assess diet starting at exam 5, the baseline visit for these analyses (1991–1995). Participants were followed for the development of cancer until 2013. The final study sample included a total of 2966 individuals, as shown in Supplementary Figure S1. A total of 3712 participants attended examination visit 5. Of these, we excluded the following participants: (a) missing or invalid FFQ data (*n* = 362); (b) history of cancer or prevalent cancer except non-melanoma skin cancer (*n* = 147); (c) aged less than 30 years at baseline (*n* = 4); and (d) missing covariates (*n* = 233). The Framingham Offspring Study data collection and these analyses were approved by the Institutional Review Board of Boston University School of Medicine (Protocols H-32086 and H-32132).

### 2.2. Dietary Assessment and Adherence to the MSDP

Diet data was assessed by self-report using a semi–quantitative food frequency questionnaire (FFQ) [18]. The FFQ includes a list of 126 food items and assesses frequency of consumption of each food during the previous year, with responses ranging from “never or <1 servings/month” to “≥6 servings/day.” Separate questions queried types of breakfast cereals and cooking oils consumed. 

The scoring methods for the MSDP score were based on adherence to a traditional Mediterranean diet [19] and have been previously described [17]. It includes data on the recommended intake of the following 13 food groups in the Mediterranean diet pyramid: wholegrain cereals, fruit, vegetables, dairy, wine, fish, poultry, olives/legumes/nuts, potatoes, eggs, sweets, meat, and olive oil. Foods from an American diet pattern that were similar to those in a traditional Mediterranean diet were also included. For example, yams are not part of the potato group in a traditional Mediterranean diet but were included in the potato category in the MSDP. The use of olive oil was scored as follows: (a) used exclusively (score = 10), (b) used olive oil and other vegetable oils (score = 5), or (c) used no olive oil (score = 0). All other foods were scored from 0 to 10, based on percent adherence to Mediterranean Diet Pyramid recommendations (e.g., consuming 80% of the recommended amount for a food category yielded a score of 8). This MSDP includes a penalty for overconsumption as well as underconsumption (e.g., exceeding the maximum recommended intake by 30% would result in a score of 7). The maximum penalty was 10 points. The total of the 13 component scores was standardized to a scale of 0–100 and weighted (from 0 to 1) by the percent of total energy derived from consuming foods included in the Mediterranean diet pyramid. For example, if 45% of energy was derived from foods not included on the Mediterranean diet pyramid, the calculated weight was 0.55. The final MSDP score ranged from 0 to 100. 

### 2.3. Cancer Outcomes

The primary occurrence of cancer was adjudicated using a standardized Framingham Study protocol as previously described [20]. Briefly, possible cancer cases were initially detected using self–report at each examination visit, surveillance of local hospital admissions, and searches of death records from the state health department and the National Death Index [21]. Framingham investigators gathered pathology reports and other clinical and laboratory data for each subject [22] for the purpose of establishing the correct cancer diagnosis and date of diagnosis [23]. In these analyses, there were 611 cancer cases identified as first primary malignant cancers; the topography and morphologic characteristics of each cancer were coded based on the International Classification of Diseases for Oncology code (ICD-O-3 only). Non–melanoma skin cancer cases were excluded in these analyses.

### 2.4. Potential Confounders and Effect Modifiers

Height and weight were measured with a standard beam balance scale with the subject wearing a hospital gown and no shoes [23]. To reduce random error and the effect of natural height loss occurring after the age of 60, we calculated each participant’s average height from all exam visits up until the age of 60 years [24]. We used this average height in combination with baseline weight at exam 5 to calculate baseline body mass index (BMI) (i.e., weight (kg) at exam 5 divided by mean height (m^2^).

Physical activity was assessed at exams 4 (1987–1991) and 7 (1998–2001) using self-report questionnaires that included recreational activities and household work. The intensity levels for light, moderate, and vigorous activities were derived from previous studies of oxygen utilization for a given level of activity. A weighted moderate and vigorous activity score was calculated by summing total hours of moderate activity (multiplied by its intensity value) and total hours of vigorous activity (multiplied by its intensity score) [25]. 

The number of years of education was self-reported at exam two and was used to classify baseline education level into three categories: high school or less, some college, and college or graduate degree. Missing education data at exam two were imputed hierarchically as follows: education level at exam 8 (2005–2008), median years of education level for the subjects with the same occupation at exam 7 (1998–2001), and sex-specific median years of education at exam 2 (1979–1983). Missing data for self-reported pack-years of cigarette smoking at exam 5 (1991–1995) were substituted with the mean of pack-years from exams 4 (1987–1991) and 6 (1995–1998) when available. Other self-reported covariates at exam 5 (1991–1995) included: multivitamin use, other supplement use, cigarette smoking status (never, former, or current smoking defined as ≥1 cigarettes per day), energy intake, and alcohol intake (g/day). Measures of abdominal adiposity, including waist circumference (cm) and waist–to–height ratio (missing values substituted using the mean from exams 4 and 6), were also collected at baseline. Further, we created a time-dependent variable to reflect self-reported estrogen use (including estrogen use only). We classified women as never or ever users of estrogen based on self-report across multiple exam visits. Type 2 diabetes was defined at baseline when the participant met one of the following criteria: (a) 10-hour-fasting glucose of ≥126 mg/dL; (b) non-fasting glucose of ≥200 mg/dL; (c) confirmed treatment of diabetes, or (d) self-reported diagnosis of possible diabetes at one visit with a subsequent diagnosis of definite diabetes at the next exam (in the absence of an excessive weight gain of 7% or more of body weight).

### 2.5. Statistical Analysis

Sensitivity analyses were based on the exposure distribution and power considerations and used to explore different cut-off values for categorizing the MSDP score. The optimal classification was selected as follows: low: 4.0–19.0 (reference group), moderate: 19.1–25.0, and high: 25.1–50.9. Incidence rates for total cancer were computed by dividing the number of cancer cases by total person-years (py) of follow-up calculated from baseline (exam 5, 1991–1995) to the first of the following events: primary occurrence of cancer, loss to follow-up, date of last exam or death. Age and multivariable-adjusted Cox proportional hazards regression models were used to estimate hazard ratios (HRs) and 95% confidence intervals (CIs) for incident cancer. A test for linear trend across MSDP score categories was performed. 

Because the effects of adherence to the MSDP may depend on the level of other lifestyle and anthropometric measures, we chose to assess interaction between MSDP adherence and each of the following risk factors: BMI, WHtR, alcohol intake, and cigarette smoking status. To better assess public health importance, interaction was assessed on an additive rather than a multiplicative scale [26,27]. Cox-models were used to estimate the relative excess risk due to additive interaction in men and women separately [28]. To optimize statistical power for these analyses, we used sensitivity analysis to dichotomize the MSDP score as ≤19 (low) vs. >19 (moderate/high). For these analyses, BMI was categorized as <25 vs. ≥25 kg/m^2^ for women and <30 vs. ≥30 kg/m^2^ for men [21,23]. The WHtR was classified as <57 vs. ≥57 for women and<51 vs. ≥51 cm/m for men [22]. Three categories of current alcohol intake were chosen to represent non-drinkers, light-to-moderate drinkers, and heavy drinkers: 0 g/day, 0.1–13.99 g/day, and ≥14.00 g/day, respectively, for men and 0 g/day, 0.1–6.9 g/day, and ≥7 g/day, respectively, for women. Cigarette smoking status was classified as current smokers, former smokers, and non-smokers. 

The final multivariable models adjusted for confounders that were found to alter the age-adjusted hazard ratios by approximately 10% or more in men and women separately. Those factors retained in the final models included BMI (kg/m^2^), cigarette pack-years, physical activity (metabolic equivalent hours/day), prevalent diabetes, and supplement use (never vs. ever). Factors not included in the final models were educational level, alcohol intake, and estrogen use because they were not found confounding the association between MSDP adherence and total cancer risk. 

Finally, we analyzed the association between each food group considered in the MSDP score and total cancer risk. Due to substantially right-skewed data, we excluded those with the highest 1% of intake for each food group and then used sensitivity analyses to determine the cut-off values to define intake as low (reference), moderate, or high for each food group. The multivariable Cox models included the same factors listed above plus total energy intake; these models also mutually adjusted for the intakes of all other MSDP score components. A test for linear trend across each food group was performed based on the category-specific medians of food intake. No violations of the proportional hazard assumptions were found in any of the models. The statistical analyses in this study were conducted using SAS statistical software, version 9.4 (SAS Institute, Cary, NC, USA).

## 3. Results

The MSDP score at baseline was normally distributed, with a mean of 22.4 (± 7.3) (range 4.0–51.0). Table 1 shows the subject characteristics according to MSDP score categories (low, moderate, high). Compared with participants in the lowest category, those with the highest scores were older, more likely to be women, had higher educational levels, slightly lower alcohol intakes, and more often used dietary supplements. In addition, they were also less likely to be current smokers. Women in the highest MSDP score category were more likely to be estrogen users.

Table 2 shows the median intakes (servings per day or per week) for each of the 13 food groups considered in the MSDP score along with their MSDP scores. Median scores were highest for poultry, potatoes, dairy, and fruits. The lowest scores were for the categories of sweets, meat, olive oil use, and wine consumption.

Of the 2966 men and women at baseline, 611 subsequently developed cancer (Table 3). Overall, those with higher MSDP scores had lower cancer incidence rates (1160 cases/10,000 py of follow-up) compared to those with moderate and lower MSDP scores, 1240 and 1416 cases/10,000 py, respectively. In the fully adjusted models, participants with moderate and higher MSDP scores had non-statistically significant 15% and 16% lower risks of total cancer than those in the lowest MSDP score category, respectively (HR_moderate_: 0.85, 95% CI: 0.70–1.04; HR_high_: 0.84, 95% CI: 0.68–1.03). In sex-stratified analyses, greater adherence to a Mediterranean diet had a much stronger beneficial effect on total cancer risk in women than in men. For women, those in the moderate and higher MSDP score categories had 29% (95% CI: 0.52–0.97) and 26% (95% CI: 0.55–0.99) lower cancer risks than those in the lowest MSDP score category. 

**Figure 1:**
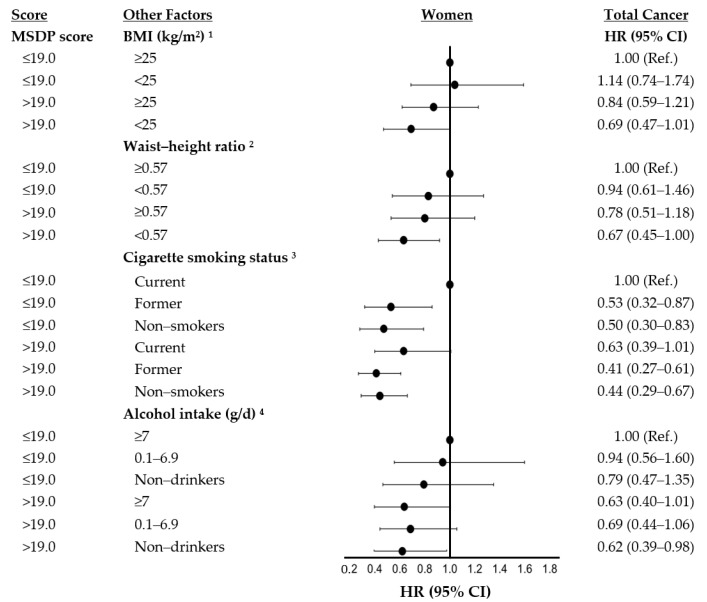
Independent and combined effects of MSDP adherence and anthropometric, lifestyle, and dietary factors on total cancer risk in women of the Framingham Offspring Study. ^1^ Models assessing effect modification by BMI were adjusted for age, pack-years of smoking, physical activity, prevalent diabetes, and supplement use. ^2^ Models assessing effect modification by waist-to-height ratio were adjusted for age, hip girth, pack-years of smoking, physical activity, prevalent diabetes, and supplement use. ^3^ Models assessing effect modification by smoking status were adjusted for age, BMI, physical activity, prevalent diabetes, and supplement use. ^4^ Models assessing effect modification by alcohol intake were adjusted for age, BMI, pack-years, physical activity, prevalent diabetes, and supplement use. For these models, wine was excluded from the MSDP score. MSDP, Mediterranean style dietary pattern; Ref, reference category; BMI, body mass index; and D, day.

**Figure 2:**
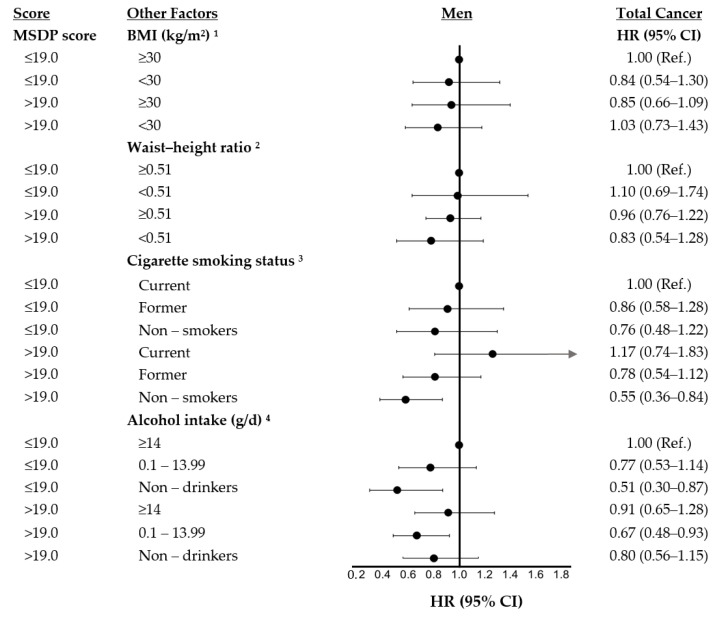
Independent and combined effects of MSDP adherence and anthropometric, lifestyle, and dietary factors on total cancer risk in men of the Framingham Offspring Study. ^1^ Models assessing effect modification by BMI were adjusted for age, pack-years of smoking, physical activity, prevalent diabetes, and supplement use. ^2^ Models assessing effect modification by waist-to-height ratio were adjusted for age, hip girth, pack-years of smoking, physical activity, prevalent diabetes, and supplement use. ^3^ Models assessing effect modification by smoking status were adjusted for age, BMI, physical activity, prevalent diabetes, and supplement use. ^4^ Models assessing effect modification by alcohol intake were adjusted for age, BMI, pack-years, physical activity, prevalent diabetes, and supplement use. For these models, wine was excluded from the MSDP score. Participants (*n* = 4, men) exceeding 100 g of alcohol per day were excluded from this model. MSDP, Mediterranean style dietary pattern; Ref, reference category; BMI, body mass index; and D, day.

Figure 1 and Figure 2 show results of analyses exploring whether the risk estimates for total cancer were modified by anthropometric measures of body fat, cigarette smoking, or alcohol use in women and men. In each of these analyses, we explored the effects of a higher (≥19) vs. lower (<19) MSDP score combined with categories (some of which are sex-specific) of a second risk factor: For example, for BMI, we classified subjects into one of the following four categories: (1) low MSDP and high BMI (ref group), (2) low MSDP and low BMI, (3) high MSDP and high BMI, and (4) high MSDP and low BMI. Those in the fourth category were hypothesized to have the lowest total cancer risk. Among women, the HRs for categories 2, 3, and 4, respectively, were 1.14 (95% CI: 0.74–1.74), 0.84 (95% CI: 0.59–1.21), and 0.69 (95% CI: 0.47–1.01), suggesting that those with a higher MSDP score and a lower BMI did in fact have the lowest risk of cancer and that protective effect appeared more than additive. Similarly, women with higher MSDP scores who also had a lower WHtR had lower cancer risks than those with either risk factor alone. However, the excess risks due to the interaction of these adiposity factors and MSDP adherence were not statistically significant (p-interaction > 0.05).

We also explored possible effect modification on an additive scale of the MSDP scores by cigarette smoking and alcohol consumption. Non-smoking and former-smoking women generally had lower cancer risks, and these associations were slightly stronger among those with higher MSDP scores. In men, we found that those with higher MSDP scores who were non-smokers had the lowest risk of cancer (HR: 0.55; 95% CI: 0.36–0.84). Once again, while these effects were more than additive, the estimated interaction did not reach statistical significance (p-interaction > 0.05). Finally, there was no consistent evidence of effect modification by alcohol intake in either men or women. 

Table 4 shows the sex-specific associations between categories of intake in each MSDP food group and total cancer risk. Compared with lower intakes, moderate intakes of some foods were associated with lower cancer risks, but these risks tended not to be statistically significant. For example, moderate dairy consumption was associated with a reduced risk of cancer in men (HR: 0.69, 95% CI: 0.51–0.94) and women (HR: 0.67, 95% CI: 0.47–0.96), but the p-values for trend were not statistically significant. Additionally, women with moderate (vs. lower) intakes of fruit, eggs, and potatoes and those who used olive oil tended to have lower cancer risks, and men with moderate intakes of potatoes tended to have lower risks. 

## 4. Discussion

This is the first long-term population-based study using the MSDP score developed by Rumawas et al. [17] to assess the association between adherence to a Mediterranean-style diet and overall cancer risk. We observed that consuming a diet consistent with the principles of the Mediterranean diet was associated with reductions in total cancer risk among healthy adults aged 30 years or older but especially among women. Those women with moderate and higher MSDP scores had at least a 26% lower cancer risk. Further, non-overweight women with a higher MSDP score had a much lower risk of cancer than any other group of women. Among men, the effects of a Mediterranean-style diet were modified by smoking status in that non-smoking men with higher adherence to a Mediterranean-style diet had the lowest cancer risks. This study shows that the effects of a Mediterranean-style diet on cancer risk appear to be modified by other risk factors. 

Only two previous studies have reported beneficial effects of the Mediterranean diet on total cancer incidence [14,15]. The multicenter EPIC study followed 142,605 men and 335,873 women for a median of 8.7 years and found that the highest Mediterranean diet score category was associated with 7% reductions in total cancer risk in both men and women [15]. These risk reductions were similar among participants in Mediterranean and non-Mediterranean countries, suggesting that the cancer-protective effects of this dietary pattern are not unique to countries in the Mediterranean region. We had insufficient power to detect statistically significant risk reductions of that magnitude in men in this current study, while the effect estimates (9% lower cancer risk associated with higher MSDP scores) were similar.

Our results suggested a stronger risk reduction in Framingham women than was observed in the larger EPIC study [15]. It is possible that the different approaches to scoring adherence to the Mediterranean diet might explain some of these differences in the observed effects. Specifically, the EPIC study used a diet score [16] with cut-off values for adequate intakes in European countries based on sex-specific population median food and nutrient intakes in those countries rather than intakes recommended by the Mediterranean diet pyramid (as was done in the current study). It may be that the lower baseline risk in the EPIC study led to weaker risk reductions in those countries. Other differences between the two studies included the addition of total alcohol in the score rather than red wine alone, as well as between-country differences in the food sources of nutrients such as monounsaturated fatty acids (MUFAs). 

A separate analysis of data from the Greek cohort in the EPIC study found that adherence to a Mediterranean diet was associated with a statistically significant 22% lower risk of cancer overall, with stronger effects found among women (a statistically significant 27% lower risk) than among men, who had a non-statistically significant 17% lower risk [14]. It is possible that these stronger effects in the Greek EPIC cohort may have been due to the median-based scoring system better capturing Mediterranean diet adherence in that population than it did in more northern European countries. Our findings using the MSDP score were similar to the sex-specific differences found in the Greek EPIC cohort. 

Excess body fat, including overweight, obesity, and weight gain (during middle adult years), are strong modifiable risk factors for certain cancers such as breast (post-menopausal), colorectal, and reproductive cancers [5,29]. The most common cancers among FOS participants were obesity-related cancers, mainly breast (post-menopausal) and colorectal. Prior analyses in the FOS study showed that gaining ≥0.45 kg (≥1.0 pound) per year over ~14 years of follow-up during the middle-adult years increased the risk of overweight and obesity-related cancers by 38% (95% CI: 1.09–1.76) [23]. Data from the Nurses’ Health Study and the Health Professional Follow-Up Study [30] as well as the Women’s Health Initiative [31] also demonstrated that weight gain increased the risk of obesity-related cancers in women. Few studies have examined effect modification of the relation between a Mediterranean diet and cancer risk by baseline BMI [32,33], and results have been inconsistent. The current study in which the effects of a Mediterranean-style diet were stronger among leaner women differ from those of some studies in which the protective effects of the Mediterranean diet (e.g., on breast cancer risk) were found mainly among obese women [34,35].

In this study, the beneficial effect of a higher MSDP score in men was strongest among never-smokers, while in an earlier EPIC study, the beneficial effects of a Mediterranean-style diet were slightly stronger among current smokers than past or never smokers [15]. It is possible that these conflicting results may be due to differences in the baseline prevalence of cigarette smoking as well as rates of tobacco-related cancers between studies or that patterns of smoking may be differently associated with other behavioral risk factors in different studies. 

An analysis of each food group considered in a Mediterranean diet score in association with total cancer risk has only been done once before. Our results showed that there was some tendency for certain food groups to be associated with lower cancer risks, especially in women, but none had statistically significant linear trends. In the earlier EPIC study, there was no evidence of an association between any individual component of the Mediterranean diet score with risk of total cancer [15]. The cancer-protective effect of the overall Mediterranean diet pattern is likely to be stronger than that of individual foods and nutrients associated with the diet pattern. The unique matrix of foods and nutrients as part of a Mediterranean dietary pattern may act synergistically to protect against the occurrence or spread of certain cancers [15]. Overall, the Mediterranean diet is rich in phytochemicals including polyphenols (such as flavonoids and resveratrol), carotenoids, and fiber, and its fatty acid profile is high in omega-3 and MUFAs. This overall nutrient profile is thought to have antioxidant, anti-inflammatory, antiproliferative, and antimutagenic properties [4,5,6,7,13,36,37,38]. 

In addition, the beneficial effects of the Mediterranean diet could be explained by intermediate effects on body fat or body composition resulting from the obesity-protective effect of the Mediterranean diet [5,10,39]. Despite the higher fat content of the Mediterranean diet, clinical and epidemiological studies showed that this diet has been linked with a low-to-moderate weight loss and lower abdominal adiposity [40,41,42]. A previous prospective analysis of individuals without diabetes mellitus in the FOS found that higher (vs. lower) MSDP scores were associated with lower waist circumference, less insulin resistance, and lower fasting plasma glucose and triglycerides, as well as higher HDL cholesterol [42]. One clinical trial demonstrated that people with metabolic syndrome assigned to a Mediterranean diet group had a 2.0 cm greater decrease in waist circumference and 2.8 kg greater weight loss than those in the control group (prudent diet) [40]. Further, central adiposity and metabolic dysfunction are both risk factors for cancer development. Therefore, a Mediterranean-style diet may result in less adiposity and metabolic dysfunction, thereby explaining in part the cancer-protective effects of this diet pattern. 

The strengths of this study include its prospective design with carefully adjudicated cancer outcomes using standardized procedures [20]. In addition, most potential confounding or effect modifying variables were measured rather than self-reported in this study. In terms of limitations, because the score that we used assigned equal weight to each component, it assumes that the biological effects of these components are all equal with respect to different types of cancer, and this may not be the case. Another limitation is the limited distribution and relatively low average MSDP scores in this study. The highest score observed was 50.95, and the mean score was less than half that amount. Further, the FFQ used to calculate the MSDP score has a limited ability to estimate energy intake, thus making it a possible source of error. However, earlier validation studies of the FFQ used in these analyses found that many of the foods included in the MSDP score were adequately measured when compared with intakes from diet records [43]. A further limitation of this study is its sample size. The relatively small numbers of cancer cases gave us limited power to evaluate individual cancers, particularly for men and women, separately. This was especially problematic in the assessment of effect modification. Similarly, the food group analyses should be interpreted with caution due to the limited number of cases in some categories of intake and the strong correlations between different food groups in these analyses. Finally, the FOS subjects were exclusively Caucasian, so these results may not be representative of risks in a multiethnic population. 

## 5. Conclusions

This is the first prospective cohort study to show that consumption of a Mediterranean-style diet may be one effective strategy for reducing total cancer risk in the US. In this study, the cancer-protective effects of higher Mediterranean diet adherence were strongest in women with less adiposity and among men who did not smoke. Given the high rates of cancer in the US [44,45], these findings have the potential to benefit large numbers of people. 

## Figures and Tables

**Table 1:** Baseline characteristics according to MSDP score categories in the Framingham Offspring Study.

	**MSDP Score Categories**		
	**Low**	**Moderate**	**High**
	**(4.0–19.0)**	**(19.1–25.0)**	**(25.1–50.9)**
**Characteristic (*n* = 2966)**	***n* = 995**	***n* = 951**	***n* = 1020**
**Sex**, *n* (%)			
Women	443 (45)	505 (53)	630 (62)
Men	592 (56)	446 (47)	390 (38)
**Age** (years)	52.8 (± 9.5)	55.0 (± 9.9)	55.2 (± 9.3)
**Age at diagnosis** (years)	70.3 (± 8.9)	72.5 (± 9.4)	73.0 (± 8.9)
**Education**, *n* (%)			
≤High School	445 (44.7)	351 (36.9)	326 (32.0)
Some college	265 (26.6)	283 (29.8)	316 (31.0)
College, Graduate degree	285 (28.6)	317 (33.3)	378 (37.1)
**BMI** (kg/m^2^)	27.4 (± 5.0)	27.4 (± 4.8)	27.1 (± 5.0)
**Waist** (cm)	93.8 (± 14.2)	93.0 (± 14.1)	91.1 (± 14.3)
**Waist-to-height ratio**	0.55 (± 0.08)	0.55 (± 0.08)	0.55 (± 0.08)
**Cigarette smoking**, *n* (%)			
Never	311 (31.3)	325 (34.3)	416 (40.8)
Former	406 (40.8)	450 (47.4)	478 (46.9)
Current	378 (27.9)	174 (18.3)	126 (12.3)
**Pack years of smoking** ^1^	20.0 (± 0.9)	16.1 (± 0.8)	12.0 (± 0.6)
**Energy intake** (kcals/day)	1741 (± 650)	1885 (± 611)	1963 (± 580)
**Alcohol** (g/day) ^1^	8.0 (± 0.4)	7.3 (± 0.3)	6.7 (± 0.3)
**Supplement use**, *n* (%)			
No	735 (73.9)	680 (71.5)	636 (62.4)
Yes	242 (24.3)	250 (26.3)	367 (36.0)
**Physical activity** (METs/day)	14.3 (± 9.2)	14.5 (± 8.1)	15.1 (± 8.0)
**Prevalent diabetes**, *n* (%)	59 (5.9)	65 (6.8)	79 (7.8)
**Estrogen use**, *n* (%) ^2^			
Never	379 (85.8)	426 (84.4)	507 (80.1)
Ever	63 (14.2)	79 (15.6)	122 (19.4)

Values are expressed as mean (±SD) or n (column percentage) or otherwise stated. ^1^ Values are expressed as geometric mean ± SE. ^2^ Sample includes 1576 women as 2 were missing estrogen use data. MSDP, Mediterranean-style dietary pattern; BMI, body mass index; D, day; and METs, Metabolic equivalents.

**Table 2:** Intake and score distributions of the food groups considered in the MSDP score among the participants in the Framingham Offspring Study.

Food Groups Considered in the MSDP Score	Median Intakes (5%–95%)	Median Scores (5%–95%) ^1^
	**Servings/day**	
Whole grains	0.90 (0.00–3.60)	1.13 (0.00–4.50)
Fruit	1.50 (0.20–4.40)	4.67 (0.33–9.33)
Vegetables	2.40 (0.80–5.80)	4.00 (1.17–8.50)
Dairy	1.20 (0.20–4.10)	5.00 (0.00–9.00)
Wine: Men	0.10 (0.00–0.90)	0.33 (0.00–3.00)
Women	0.10 (0.00–0.90)	0.67 (0.00–5.33)
	**Servings/week**	
Olives, pulses, and nuts	1.20 (0.00–5.10)	3.00 (0.00–9.25)
Potatoes	3.50 (0.50–7.60)	5.00 (0.00–10.00)
Poultry	5.00 (0.80–9.90)	5.75 (0.00–8.25)
Eggs	0.50 (0.00–3.00)	1.67 (0.00–10.00)
Meat	4.00 (0.50–11.90)	0.00 (0.00–8.00)
Sweets	13.80 (1.90–45.00)	0.00 (0.00–7.67)
Fish	2.30 (0.50–7.00)	3.73 (0.67–9.00)
Olive oil use score ^2^		0.00 (0.00–10.00)

^1^ Scores ranged from 0 to 10 based on percent adherence to Mediterranean Diet Pyramid recommendations, except for the use of olive oil. ^2^ Olive oil was scored as (a) used exclusively (score = 10), (b) used olive oil and other vegetable oils (score = 5), or (c) used no olive oil (score = 0). MSDP, Mediterranean style dietary pattern.

**Table 3:** Hazard ratios for total cancer, according to MSDP score categories in the Framingham Offspring Study.

	Subjects	Cases/PY	Rate/10,000 py	HR (95% CI) ^1^	HR (95% CI) ^2^
**All subjects (*n* = 2966)**					
**MSDP score**					
Low (4.0–19.0)	995	226/15,964	1416	1.00 (Ref.)	1.00 (Ref.)
Moderate (19.1–25.0)	951	191/15,407	1240	0.82 (0.67–0.99)	0.85 (0.70–1.04)
High (25.1–50.9)	1020	194/16,717	1160	0.79 (0.65–0.96)	0.84 (0.69–1.03)
P-trend				0.02	0.09
**Women (*n* = 1578)**					
**MSDP score**					
Low (4.0–19.0)	443	88/7333	1200	1.00 (Ref.)	1.00 (Ref.)
Moderate (19.1–25.0)	505	74/8552	865	0.86 (0.50–0.93)	0.71 (0.52–0.97)
High (25.1–50.9)	630	97/10,718	905	0.70 (0.53–0.94)	0.74 (0.55–0.99)
P-trend				0.02	0.05
**Men (*n* = 1388)**					
**MSDP score**					
Low (4.0–19.0)	552	138/8632	1599	1.00 (Ref.)	1.00 (Ref.)
Moderate (19.1–25.0)	446	117/6856	1707	0.90 (0.70–1.16)	0.95 (0.74–1.22)
High (25.1–47.7)	390	97/5999	1617	0.83 (0.64–1.08)	0.91 (0.70–1.20)
P-trend				0.17	0.51

^1^ Adjusted for age and sex (for all subjects’ models). ^2^ Adjusted for age, baseline body mass index, pack-years of smoking, physical activity, prevalent diabetes, supplement use, and sex (for all subjects’ models). MSDP, Mediterranean style dietary pattern; PY, person-years; and Ref, reference category.

**Table 4:** Hazard ratios for total cancer, according to the intake of each food group considered in the MSDP score in women and men of the Framingham Offspring Study.

Food Groups Considered in the MSDP Score (servings/day)	Women	Men
	HR (95% CI) ^1^	HR (95% CI) ^1^
**Whole grains**		
Low (<0.5)	1.00 (Ref.)	1.00 (Ref.)
Moderate (0.5–<1.0)	0.90 (0.62–1.31)	1.21 (0.88–1.66)
High (≥1)	1.11 (0.79–1.55)	1.10 (0.83–1.46)
P-trend	0.39	0.66
**Fruit**		
Low (<0.75)	1.00 (Ref.)	1.00 (Ref.)
Moderate (0.75–<2.5)	0.62 (0.44–0.88)	1.10 (0.81–1.48)
High (≥2.5)	0.87 (0.56–1.34)	1.02 (0.70–1.48)
P-trend	0.92	0.93
**Vegetables**		
Low (<1.5)	1.00 (Ref.)	1.00 (Ref.)
Moderate (1.5–<3)	0.99 (0.67–1.44)	0.95 (0.71–1.27)
High (≥3)	0.81 (0.52–1.26)	1.08 (0.75–1.55)
P-trend	0.22	0.59
**Dairy**		
Low (<0.5)	1.00 (Ref.)	1.00 (Ref.)
Moderate (0.5–<1.5)	0.67 (0.47–0.96)	0.69 (0.51–0.94)
High (≥1.5)	0.73 (0.49–1.08)	1.02 (0.74–1.40)
P-trend	0.43	0.17
**Wine**		
Non–drinkers	1.00 (Ref.)	1.00 (Ref.)
Drinkers	1.07 (0.81–1.41)	1.12 (0.89–1.42)
P-trend	0.64	0.34
**Servings/week**		
**Olives, pulses & nuts**		
Low (<0.5)	1.00 (Ref.)	1.00 (Ref.)
Moderate (0.5–<1.5)	1.09 (0.74–1.63)	0.80 (0.54–1.17)
High (≥1.5)	0.89 (0.58–1.37)	0.89 (0.59–1.34)
P-trend	0.32	0.82
**Potatoes**		
Low (<1.5)	1.00 (Ref.)	1.00 (Ref.)
Moderate (1.5–<3.5)	0.73 (0.50–1.08)	0.72 (0.51–1.01)
High (≥3.5)	0.76 (0.52–1.12)	0.87 (0.63–1.20)
P-trend	0.39	0.95
**Poultry**		
Low (<2)	1.00 (Ref.)	1.00 (Ref.)
Moderate (2–<5)	1.04 (0.66–1.64)	0.76 (0.54–1.08)
High (≥5)	1.19 (0.86–1.65)	0.88 (0.67–1.15)
P-trend	0.28	0.45
**Meat**		
Low (<2)	1.00 (Ref.)	1.00 (Ref.)
Moderate (2–<5)	1.22 (0.85–1.75)	1.63 (1.11–2.42)
High (≥5)	1.14 (0.75–1.74)	1.46 (0.95–2.26)
P-trend	0.81	0.67
**Sweets**		
Low (<7)	1.00 (Ref.)	1.00 (Ref.)
Moderate (7–<21)	0.92 (0.67–1.27)	0.96 (0.70–1.34)
High (≥21)	0.79 (0.50–1.27)	0.94 (0.64–1.37)
P-trend	0.34	0.75
**Fish & other seafood**		
Low (<2)	1.00 (Ref.)	1.00 (Ref.)
Moderate (2–<4)	0.84 (0.61–1.16)	1.12 (0.86–1.45)
High (≥4)	1.18 (0.83–1.67)	0.95 (0.69–1.32)
P-trend	0.41	0.85
**Eggs**		
Low (0)	1.00 (Ref.)	1.00 (Ref.)
Moderate (0.5–<1.5)	0.69 (0.48–0.98)	0.99 (0.70–1.40)
High (≥1.5)	0.82 (0.58–1.15)	0.97 (0.70–1.34)
P-trend	0.89	0.85
**Olive oil**		
No use of olive oil	1.00 (Ref.)	1.00 (Ref.)
Olive oil use	0.71 (0.49–1.01)	0.89 (0.67–1.20)
Olive oil and Vegetable oil use	0.93 (0.47–1.84)	1.37 (0.69–2.70)
P-trend	0.06	0.51

^1^ Adjusted for age, body mass index, pack-years of smoking, physical activity, diabetes status, supplement use status, calorie intake and mutually adjusted for all the other MSDP food groups. MSDP, Mediterranean style dietary pattern and Ref., reference category.

## Data Availability

This manuscript was prepared using Framingham Offspring Research Materials obtained from the NHLBI Biologic Specimen and Data Repository Information Coordinating Center and does not necessarily reflect the opinions or views of the Framingham Offspring Study or the NHLBI. Data can be found upon request at https://biolincc.nhlbi.nih.gov/studies/framoffspring/ (accessed on 12 November 2021).

## Supplementary Materials

The following are available online at https://www.mdpi.com/article/10.3390/nu13114064/s1, Figure S1: Flowchart of study participants.
