# Supplementary figures and images for: Adherence to a Mediterranean-Style Dietary Pattern and Cancer Risk in a Prospective Cohort Study
Source: Nutrients. 2021 Nov 13;13(11):4064. doi: 10.3390/nu13114064 (PMC8622098; doi:10.3390/nu13114064)

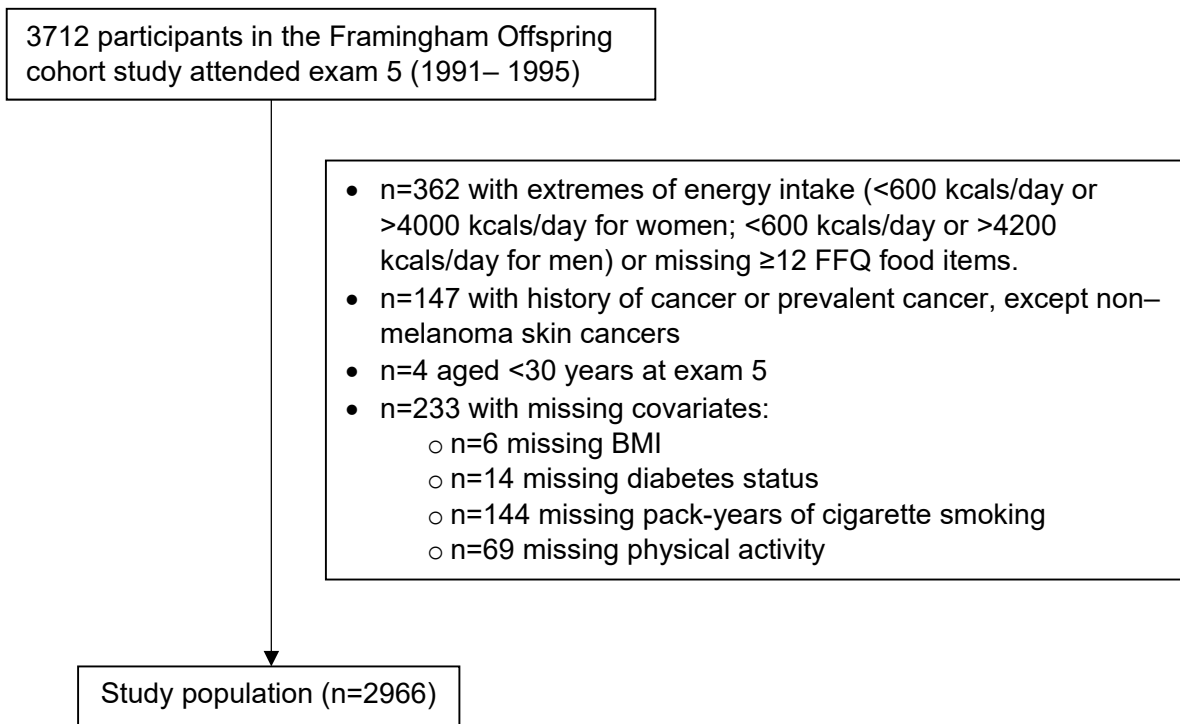

**Supplementary Figure S1.** Flowchart of study participants.

Supplement: Supplementary file 1 [file nutrients-13-04064-s001.zip › nutrients-1457011-supplementary.pdf]
